# Converging Evidence of Similar Symptomatology of ME/CFS and PASC Indicating Multisystemic Dyshomeostasis

**DOI:** 10.3390/biomedicines11010180

**Published:** 2023-01-11

**Authors:** David F. Marks

**Affiliations:** Independent Researcher, 13200 Arles, France; dfmarksphd@gmail.com

**Keywords:** Central Homeostasis Network, chronic fatigue syndrome, dyshomeostasis, hypothesis, ME/CFS, post-acute sequelae of SARS-CoV-2, PASC, myalgic encephalomyelitis, pathophysiology, symptomatology

## Abstract

The purpose of this article is to review the evidence of similar symptomatology of myalgic encephalomyelitis/chronic fatigue syndrome (ME/CFS) and post-acute sequelae of SARS-CoV-2 infection (PASC). Reanalysis of data from a study by Jason comparing symptom reports from two groups of ME/CFS and PASC patients shows a notably similar symptomatology. Symptom scores of the PASC group and the ME/CFS group correlated 0.902 (*p* < 0.0001) across items. The hypothesis is presented that ME/CFS and PASC are caused by a chronic state of multisystemic disequilibrium including endocrinological, immunological, and/or metabolic changes. The hypothesis holds that a changed set point persistently pushes the organism towards a pathological dysfunctional state which fails to reset. To use an analogy of a thermostat, if the ‘off switch’ of a thermostat intermittently stops working, for periods the house would become warmer and warmer without limit. The hypothesis draws on recent investigations of the Central Homeostasis Network showing multiple interconnections between the autonomic system, central nervous system, and brain stem. The hypothesis helps to explain the shared symptomatology of ME/CFS and PASC and the unpredictable, intermittent, and fluctuating pattern of symptoms of ME/CFS and PASC. The current theoretical approach remains speculative and requires in-depth investigation before any definite conclusions can be drawn.

## 1. Introduction

This narrative review is concerned with the nature of two similar life-threatening disorders: ‘ME/CFS’ (a term referring to two highly similar disorders named ‘chronic fatigue syndrome’ or ‘CFS’ and ‘myalgic encephalomyelitis’ or ‘ME’, combined here into a single category); and post-acute sequelae of SARS-CoV-2 infection or ‘PASC’ (a shortened name given to the post-acute form of SARS-CoV-2 infection, or Coronavirus disease 2019 (COVID-19). It is speculated that the post-viral pathogenesis of PASC may be similar to, or even identical to, that of ME/CFS [1]. A number of researchers have proposed pathophysiological mechanisms that may account for the similarities between ME/CFS and PASC [1,2,3,4,5,6]. The aims of this article are: (i) to indicate the converging evidence that ME/CFS and PASC involve a highly similar constellation of symptoms; (ii) to propose the hypothesis that ME/CFS and PASC have a similar etiology founded on a breakdown of the body’s central systems of homeostasis; and (iii) to indicate the possibility that neuroinflammation may be responsible for the dyshomeostasis that occurs in ME/CFS and PASC.

ME/CFS is a serious, unpredictable, complex, multisystem, chronic illness that can profoundly limit the health, activities, and psychosocial wellbeing of affected patients. The condition is estimated to affect approximately 70 million people worldwide with a prevalence of 0.89% according to the Centers for Disease Control 1994 case definition, with women approximately 1.5 to two times higher than men in all categories [7]. The US prevalence and economic impact estimates of the 2015 National Academy of Medicine report on ME/CFS were updated taking into account population growth, economic inflation, and inclusion of children [8]. A doubling of ME/CFS prevalence to 1.5 million (0.45%) was reported and an economic impact in the US in the range of 36–51 billion dollars per year. The figures for PASC as a highly transmissible infectious respiratory illness exist in two main stages, acute and post-acute (or chronic). The acute phase lasts between seven and 28 days with an estimated 10–30% of patients developing PASC. It has been estimated that the global pooled prevalence of PASC is 0.43 (95% confidence interval, 0.39–0.46) with women being around 1.3 times more likely than men to fall ill with PASC [9]. An estimated 200–250 million individuals are likely to be affected by PASC with high impact on health care systems worldwide. Combining the two populations together indicates that an estimated total of 270–320 million people could have ME/CFS or PASC, i.e., between one-in-25 to one-in-30 of the world population.

Here I review the converging evidence that ME/CFS and PASC involve a highly similar pattern of symptoms. This finding is compatible with the hypothesis that the two illnesses have a similar etiology. I offer the hypothesis that the disorders of ME/CFS and PASC are both produced by a breakdown in the body’s Central Homeostasis Network in which one or more changed set point(s) persistently push(es) the organism towards a pathophysiological state, which fails to normalize. To use an analogy of a thermostat, it is as if the ‘off switch’ of a thermostat intermittently stops working and the house becomes warmer and warmer without limit. Before discussing the specific details of the hypothesis, it is helpful to review the symptoms of each condition in turn and then to consider a controlled study in which the symptom profile of patients from the two clinical groups are directly compared and correlated.

## 2. ME/CFS

There is no agreed scientific explanation of the disorders, no diagnostic marker, no treatment, and no cure. For present purposes, the two conditions labelled ‘ME’ and ‘CFS’ will be considered as ‘ME/CFS’. Although the etiology of ME/CFS is unknown, in many cases, symptoms may be triggered by an infection or other prodromal event, such as ‘immunization, anaesthetics, physical trauma, exposure to environmental pollutants, chemicals and heavy metals, and rarely blood transfusions’ [10,11].

A discredited, yet influential, psychiatric theory of ME/CFS suggests that this a post-viral psychosomatic condition based on dysfunctional beliefs, deconditioning, and biased attention [12]. A recent review suggests that the psychosomatic approach is unsupported by empirical evidence; treatments based on the approach are ineffective, produce stigmatization and disbelief of patients, and often cause patient harms [13]. The psychosomatic approach has neglected the burgeoning literature on the biological mechanisms of ME/CFS and, arguably, delayed progress in the scientific understanding of ME/CFS by 30 years. The current medical ignorance regarding the etiology of ME/CFS has been highlighted with the recent emergence of PASC, which has many similarities and overlaps with ME/CFS.

The Institute of Medicine’s (IOM’s) [10] diagnostic criteria for ME/CFS are shown in Table 1.

Other ME/CFS manifestations listed by the IOM [10] are:

- Pain—very common, but highly variable in presence, nature, and severity
- Certain infections may act as triggers
- Gastrointestinal and genitourinary problems
- Sore throat or scratchy throat
- Painful or tender axillary/cervical lymph nodes
- Sensitivity to external stimuli

Substantial decrease or impairment in function with profound fatigue reported by the IOM included patient descriptions such as the following:

- ‘Flu-like fatigue/exhaustion’
- ‘I feel like a battery that is never able to be recharged fully despite resting a lot and limiting my activities to only the bare essentials needed to get by’
- ‘Thinking takes a lot more work than it used to’
- ‘My arms, legs, body feel heavy and harder to move’
- Severe limitations in personal and household management
- Loss of job, medical insurance, and career
- Being predominantly housebound
- Decreased social interaction and increased isolation

Of key importance, ‘post exertional malaise’ (PEM) is a prolonged exacerbation of a patient’s baseline symptoms after physical/cognitive/orthostatic exertion or stress. PEM may be delayed relative to the trigger. Patient descriptions include: ‘crash,’ ‘relapse,’ ‘collapse,’ being mentally tired after the slightest effort, and being physically drained or sick after mild activity. The more demanding, prolonged, or repeated the activity, the more severe and prolonged the payback. Other important symptoms include: unrefreshing sleep (feeling unrefreshed after sleeping many hours), cognitive impairments (problems with thinking exacerbated by exertion, effort, or stress or time pressure), and orthostatic intolerance (symptoms worsen upon assuming and maintaining an upright posture and are improved, though not necessarily abolished, by lying back down or elevating feet [10] (IOM, 2015).

## 3. PASC

**Figure 1:**
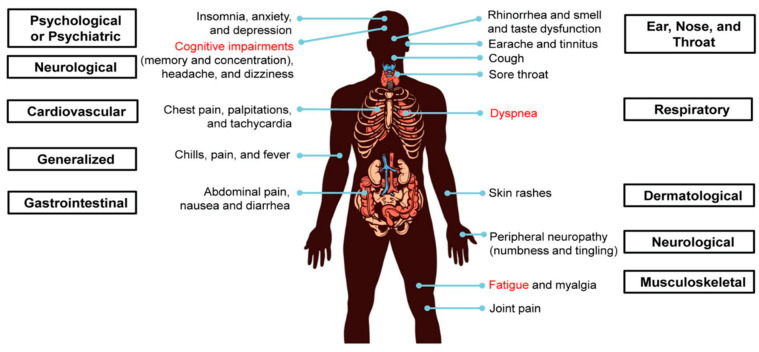
Symptoms of long-COVID-19. Multiple organ systems are affected, such as psychological or psychiatric (insomnia, anxiety, and depression), neurological (cognitive impairments involving memory and concentration, headache, dizziness, and peripheral neuropathy), ear, nose, and throat (rhinorrhea, smell and taste alterations, earache, tinnitus, cough, and sore throat), respiratory (dyspnea and cough), cardiovascular (chest pain, palpitations, and tachycardia), gastrointestinal (abdominal pain, nausea and diarrhea), generalized (chills, pain, and fever) and musculoskeletal (fatigue, myalgia, and joint pain). Dyspnea, fatigue, and cognitive impairments are among the most common symptoms. Reproduced from [14] under Creative Commons.

PASC is characterized by persistent symptoms of fatigue, insomnia, dyspnea, dizziness, chest pain, sensory deficits, and cognitive impairments lasting for six months or more after the acute phase of COVID-19 (Figure 1).

## 4. Controlled Study of PASC and ME/CFS Symptomatology

It is possible to directly compare the symptom profile of PASC and ME/CFS patients using a psychometrically validated symptom questionnaire. The following analysis uses public domain data from a study of symptoms among 278 PASC patients at two points of time and 502 patients diagnosed with ME/CFS [15]. The PASC sample was recruited in 2020 on social media sites, dedicated to the exchange of information among ‘long-haulers’. Participants were asked to complete two symptom questionnaires at one time point, with one describing current symptoms and one recounting experiences from an average of 21.7 weeks prior. The ME/CFS sample was collected by the Solve ME/CFS Initiative (https://solvecfs.org, (accessed on 10 January 2022)). All participants were recruited by a physician and were previously diagnosed with ME/CFS by a specialist [15]. The ME/CFS group was on average 10 years older, with a lower proportion of females (76.9 vs. 84.2%) and a higher proportion of white individuals (97.7 vs. 87.0%) compared to the PASC group. The illness duration of the COVID-19 sample was a mean of 21.7 weeks, significantly lower than the ME/CFS sample, 13% of whom reported being ill since childhood and, of the others, 85% had been ill for over two years [15].

The data for the study were collected using the *DePaul Symptom Questionnaire (DSQ)*, which consists of 54 self-report items, to measure ME/CFS symptomatology, demographics, medical, occupational, and social history [15]. The scale provides items distributed across eight domains: sleep (six items), PEM (six items), neurocognitive (13 items), immune (five items), neuroendocrine (eight items), pain (five items), gastrointestinal (five items), and orthostatic (six items). A fine discrimination of symptoms is possible using the DSQ, e.g., the sleep domain contains six specific items (unrefreshing sleep, needing to nap, difficulty falling asleep, difficulty staying asleep, waking up early, sleep all day) in addition to the generic item, sleep. People with ME/CFS were asked to rate the frequency of each symptom over the past six months on a five-point Likert scale with 0 = none of the time, 1 = a little of the time, 2 = about half the time, 3 = most of the time, and 4 = all of the time. Participants were also asked to rate the severity of each symptom over the past six months on a five-point Likert scale with 0 = symptom not present, 1 = mild, 2 = moderate, 3 = severe, and 4 = very severe. Participants in the PASC group rated the DSQ symptom list as they were experienced during the first two weeks of the illness and at the current time. All frequency and severity scores were standardized to a 100-point scale. The frequency and severity scores for each symptom were averaged to create one composite score for each symptom. The DSQ has strong reliability and validity and the ability to accurately differentiate individuals with ME/CFS from individuals with other chronic illnesses [15].

**Figure 2:**
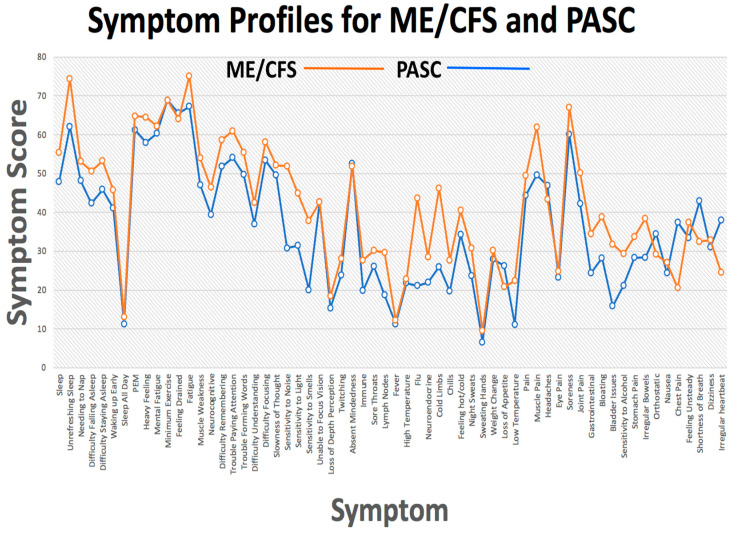
Symptom profiles for the PASC and ME/CFS patient groups. Data from [15]. Public domain.

All participants completed the 54 items of the DSQ and eight additional CDC COVID-19 symptoms giving a total of 62 items. Over time, the PASC group reported ‘an overall reduction of most symptoms including unrefreshing sleep and post-exertional malaise, but an intensification of neurocognitive symptoms. When compared to ME/CFS, the COVID-19 sample …evidenced significantly less severe symptoms than those with ME/CFS, except in the orthostatic domain’ {15}. Considered individually, the majority of items showed a significant different mean score between the two patient groups with the ME/CFS group scores at a higher level. Owing to the different illness durations and demographics of the patient groups, it is impossible to make any generalizations regarding the relative severity of symptoms between the two illnesses. However, considering the data holistically, there is notable similarity in the symptom profile of the two patient groups. An analysis conducted by this author shows that the symptom pattern for the two patient groups was highly similar (Figure 2).

**Figure 3:**
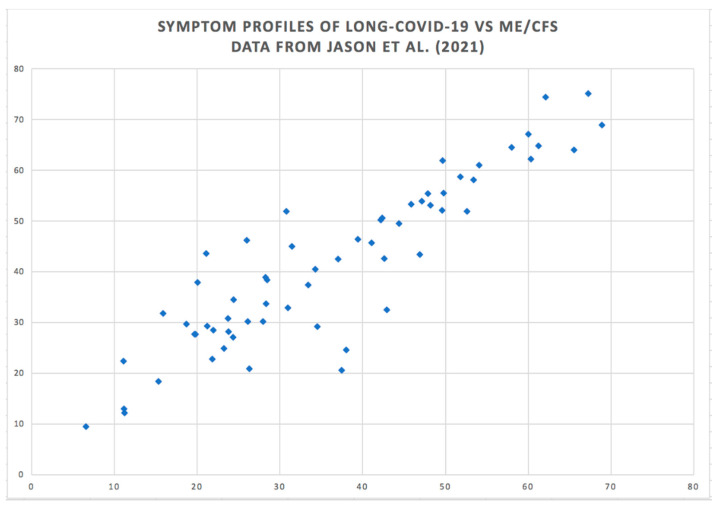
Correlation between ME/CFS and PASC symptom scores across 62 symptoms. Data from [15]. Public domain.

In another analysis, correlations across the 62 symptom scores for the group of PASC patients between times one and two was found to be 0.839 (*p* < 0.0001). Symptom scores of the PASC group at time one and those for the ME/CFS group showed a correlation of 0.729 (*p* < 0.0001). Symptom scores of the PASC group at time two and the ME/CFS group scores correlated 0.902 (*p* < 0.0001) (Figure 3). This correlation is the maximum that could be obtained within the reliability of the instrument.

Figure 2 and Figure 3 show the high degree of similarity in the profiles of symptom scores across the two conditions. Why is this the case? One explanation could be that the two disorders are different forms of a single disorder, in which case, it would be expected that the two conditions would have a similar etiology. The illnesses also share the key feature of a waxing and waning of symptoms [16,17,18]. A patient survey with 5822 ME patients in Norway reported: ‘Large fluctuations, or fluctuations with gradual deterioration, are the two most typical courses of the illness’ [19]. Another study [20] found that acute symptoms of mild to moderate COVID-19 are highly heterogeneous across individuals and over time. The waxing and waning of symptoms is equally apparent in PASC, which leaves patients with other serious conditions vulnerable [21,22]. Waxing and waning thus provides a further clue to the etiology of the two disorders.

One possible hypothesis is that these two complex conditions are both the result of a multisystemic breakdown of the body’s protective system of homeostasis. The remainder of this article is concerned with dyshomeostasis as a single parsimonious account of the nature and origin of ME/CFS and PASC. I discuss the problems that can result from systemic dyshomeostasis and specific mechanisms that have been suggested to explain the occurrence of PASC and ME/CFS.

## 5. Multisystemic Dyshomeostasis, ME/CFS and PASC

At every level of the organism, homeostasis is a drive towards stability and equilibrium. Physical and mental health rely on the continuity and reliability of corrective, restorative processes to enable the body to function normally. All physical and mental disorders are caused by disruptions accompanied by temporary or long-standing changes in homeostasis. At the most general level, the body uses three biological systems for the maintenance of equilibrium: the autonomic nervous system, the endocrine system, and the immune system. Continuous, reciprocal interaction between the nervous system, organs, and gut is essential for smooth and efficient control and coordination of bodily functions, experience, and behavior. There are multiple channels of communication. Endocrine substances directly affect the nervous and immune systems. The CNS innervates every organ and tissue of the immune system with reciprocal connections. The continuous interactions of the nervous, endocrine, and immune systems have been given the term ‘neuroimmunomodulation’ [23].The neuroendocrine and neuroimmune systems are so well integrated with the ANS that they are said to act as parts of an ‘extended autonomic system’.

**Figure 4:**
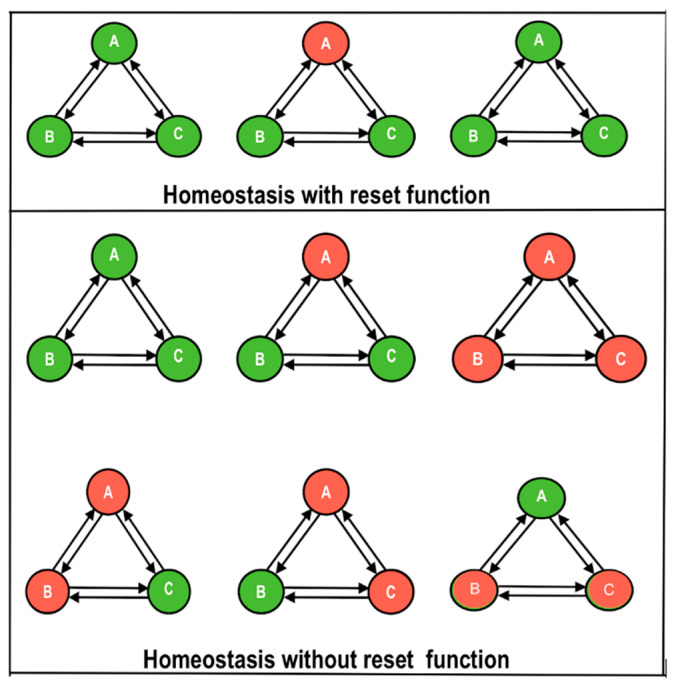
The dyshomeostasis hypothesis showing three processes or systems, A, B, and C. Upper panel: functioning normally, homeostasis resets any process that strays into disequilibrium. Upper left: A, B, and C are shown in equilibrium (green color). Upper center: A has fallen out of equilibrium (red) and requires a reset. Upper right: A is reset, and all three processes have returned to equilibrium. Lower panel: a non-resetting state of chronic dyshomeostasis is shown. Centre left: three systems A, B, and C in equilibrium (all green). Centre: A is out of equilibrium and requires a reset. Right: the system fails to reset and remains in disequilibrium. Lower left: A and B both are out of equilibrium. Lower center: A and C are out of equilibrium. Lower right: B and C are out of equilibrium. The system as a whole remains in unstable disequilibrium.

It has been suggested that aberrant homeostasis may play a role in the etiology of ME/CFS, e.g., [24,25,26]. The current hypothesis is unique in proposing that, unlike disorders that typically return the equilibrium to a previously established or fixed set point or range, *ME/CFS and PASC involve a changed set point in which the organism is pushed towards a pathophysiological state in which the disequilibrium is unable to reset.* The ‘Dyshomeostasis Hypothesis’ is illustrated in Figure 4. It can be seen that in attempting to reset a state of disequilibrium in one system, the other connected systems themselves are placed in jeopardy leading to a rotating and intermittent series of states of disequilibrium. A lack of homeostatic resetting within any one of the systems, A, B, or C, could potentially cause dyshomeostasis through the entire system (A + B + C). In the present context, A, B, and C are the nervous system, immune system, and endocrine system, respectively.

Investigators are in overall agreement regarding the symptomatology of ME/CFS and PASC with a convergence of evidence pointing to fatigue, headache, cognitive dysfunction, post-exertional malaise, orthostatic intolerance, and dyspnea as the main symptoms of both disorders [1,2,3,4,5]. What is not well understood is the intermittent ‘revolving door’ and relapsing nature of the disorders, where a patient can feel well one day but very unwell a few days later. One possible reason for the ‘revolving door’ of these illnesses is the protective relationship between inflammation and homeostasis in which inflammatory signals, such as cytokines and chemokines, can induce changes in multiple biological processes, ranging from local vascular responses to alterations in body temperature [27]. In a review of the HPA axis function in chronic fatigue syndrome, [28] found that patients with CFS normally suffer from HPA axis dysfunction. In a systematic review, [29] concluded that cytokines in blood and cerebrospinal fluid are closely associated with the progression and severity of CFS.

Inflammatory signals can directly stimulate or inhibit various homeostatic systems, change sensitivity to homeostatic signals, and change the gain of the controllers. Thus, homeostatic and inflammatory signals employ identical methods to change the same homeostatic variables. Any homeostatic system that has an adjustable set point is vulnerable to dysregulation and may run out of control. For example, the adjustable set point for body weight and adiposity allows for adaptation in times of food abundance or scarcity, as well as in the accumulation of fuel stores to feed a growing fetus [27]. In the contemporary toxic food environment, it appears likely that adjustable set points have contributed to the current obesity epidemic [30].

A review of the pathophysiology of ME/CFS has been published [31] and that of ME/CFS and PASC also [1] What kind of system can possibly explain the diverse and profound nature of ME/CFS and PASC symptoms? The following section will consider the role of a recently identified *Central Homeostasis Network* in the regulation of the body’s defenses.

## 6. Central Homeostasis Network

**Figure 5:**
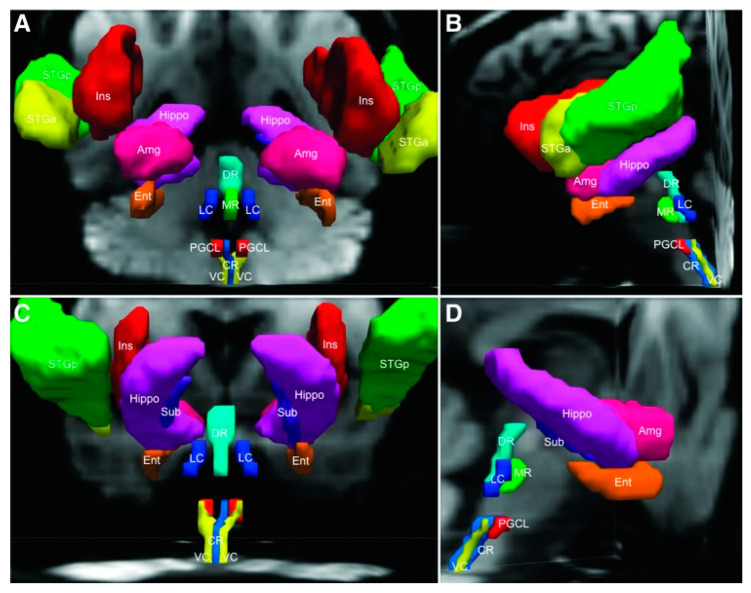
Brainstem seed regions and homeostatic forebrain regions are demonstrated with three-dimensional reconstructions in native diffusion space from an anterior (**A**), left lateral (**B**), posterior (**C**), and right lateral (**D**) perspective. Amg, amygdala; CR, caudal raphe; DR, dorsal raphe; Ent, entorhinal cortex; Hippo, hippocampus; Ins, insula; LC, locus coeruleus; MR, median raphe; PGCL, paragigantocellularis lateralis; STGa, superior temporal gyrus (anterior); STGp, superior temporal gyrus (posterior); Sub, subiculum; VC, vagal complex. Reproduced with permission from [33].

PASC and ME/CFS are debilitating, multisystemic disorders for which there exists evidence of dysregulation of the CNS, immune system, and cellular energy metabolism. All of these types of dysregulation can be linked to a hierarchical central autonomic network regulating what has been called an ‘extended autonomic system’ (EAS) [32]. Acute, coordinated alterations in homeostatic settings are believed to be crucial for surviving illnesses and other stressors. However, it is thought that intense or long-term EAS activation may cause harm because dyshomeostasis may reduce thresholds for induction of ‘destabilizing, lethal vicious cycles’ [32]. A converging view of ME/CFS is that homeostasis runs into ‘overdrive’ in which the set point remains at clinically dangerous levels for indefinite time periods and fails to reset. This undesirable condition almost certainly involves the Central Homeostasis Network, linking autonomic and cardiorespiratory nuclei in the midbrain, pons, and medulla oblongata with forebrain sites critical to homeostatic control [33]. A tractography analysis with six healthy adults revealed connections between six brainstem nuclei and seven forebrain regions, several over long distances between the caudal medulla and cerebral cortex. The strongest evidence for brainstem-homeostatic forebrain connectivity in this study was found between the brainstem midline raphe and the medial temporal lobe. A lateral forebrain bundle appears to be the primary conduit for connections between the brainstem and medial temporal lobe (Figure 5).

The findings of research on brainstem-homeostatic forebrain connectivity support the concept of an integrated Central Homeostasis Network (CHN) in the human brain [33]. The findings improve understanding of the complex and highly distributed neuroanatomic basis of homeostasis in the normal human brain, as well as for mapping CHN disconnections in patients with disorders of homeostasis, including sudden and unexpected death, epilepsy, and potentially ME/CFS and PASC as well. Neuroinflammation, which can be neurogenic, anywhere inside the CHN would appear likely to have devastating consequences [34,35,36]. To ensure replicability, significant methodological problems with neuroinflammation studies of ME/CFS need to be addressed [37].

Studies of the functional connectivity (FC) of different CNS structures has revealed differences in people with ME/CFS that are associated with severity of their fatigue symptoms. As noted, it seems possible that a breakdown in homeostatic synergy between the nervous, immune, and endocrine systems could be a significant problem in ME/CFS and PASC (Figure 4). The idea of discoordination between physiological networks is supported by multiple studies. For example, an association exists between physiological network structure and different sleep stages [38]. Circadian rhythms appear crucial to the maintenance of the sleep–waking cycle along with body-wide homeostasis. There is a possibility of a circadian breakdown in ME/CFS and PASC as a consequence of changes in transforming growth factor beta (TGFB), a cytokine previously associated with ME/CFS especially in peripheral cells [39]. Dysregulated TGFB signaling may disrupt physiological rhythms in sleep, activity, and cognition, leading to insomnia, energy disturbances, cognition problems, depression, and autonomic dysfunction.

The association between fatigue and altered resting-state FC in 36 female subjects (19 ME/CFS and 17 healthy controls) was investigated using a fatigue inventory before undergoing functional magnetic resonance (fMRI) imaging [40]. In the second of two methods, five clusters within the right parahippocampus and occipital lobes, which demonstrated significant rCBF reductions in the ME/CFS patients, were used as seeds. The degree of abnormal connectivity was found to be correlated with the level of self-reported fatigue [40]. Similar disrupted patterns of resting state FC have been observed to correlate with levels of fatigue and pain [41,42,43]. Imaging studies have also revealed abnormalities in brain structure and regional volumes [44,45,46,47,48], intra brainstem connectivity [49], (and cortical hypoactivation during resting EEG [50], including grey matter and white matter changes [45,46,47,50,51,52]. In a study of cortical autonomic network connectivity, ‘reduced higher-order homeostatic regulation and adaptability in ME/CFS’ was observed [53], which, if confirmed, would suggest the CHN as a potential therapeutic target for managing patient symptoms.

These findings converge to indicate widespread CNS involvement in ME/CFS and, by implication, PASC. Neuroinflammation could be one of the causal mechanisms for the observed abnormalities.

A few theories of ME/CFS and PASC have focused on the dysregulation of homeostasis, especially in relation to energy levels, the immune system, and neuroinflammation. The following section provides recent examples.

## 7. Examples of Dyshomeostasis in ME/CFS and PASC

The present theory is consistent with related approaches to the pathophysiology of ME/CFS. Here I consider two theories of the pathophysiology of ME/CFS and PASC that are consistent with dyshomeostasis theory. First, the ‘Energy Envelope’ theory’ [54] proposes that patients with ME/CFS need to maintain expended energy levels within the energy levels they have available. Jason’s theory found empirical support from a study in which a daily ‘energy quotient’ was established by dividing the expended energy level by the perceived energy level and multiplying it by 100. Jason predicted that participants who expended energy beyond their level of perceived energy would have more severe fatigue and other symptoms and lower levels of physical and mental functioning, which was confirmed. Jason’s theory is consistent with the current dyshomeostasis hypothesis that includes the physiological equivalent of the ‘envelope’ in the form of a set range. By maintaining certain energy levels within a set range, the patient is able to maintain homeostasis in multiple physiological systems and lower the severity of their symptoms. However, if patients exert themselves beyond that ‘envelope’, or what here we would call the set range, multiple systems could become dysregulated. This explains why patients with ME/CFS must not overexert themselves or risk severe dyshomeostasis in the form of symptoms such as post-exertional malaise. Only by maintaining energy expenditure within the set range can a patient with ME/CFS and PASC control the level and type of symptoms.

The second supportive approach is the theory of Tate and colleagues, who have discussed the role of chronic neuroinflammation and immunological dysfunction in ME/CFS [55,56] and more recently in PASC and ME/CFS [5]. Biomarkers of inflammation and leaky gut syndrome as a possible result of microbiome disturbance and bacterial translocation have been reported [55,57,58,59]. According to this theory, an aberrant state of homeostasis is the central causal mechanism of ME/CFS [25,26,55,56,60]. 

Stress is known to suppress immune function and increase susceptibility to infections and cancer [61]. Stress also influences immunity in people with chronic fatigue syndrome [62]. The immunosuppressive effects of increased cortisol on some specific immune cells, such as neutrophils and natural killer cells, have been reported [63]. Increased stress (as well as viral infections) increase cortisol, possibly leading to immunosuppression and contributing to the development of ME/CFS. In line with the typical course of the illness, it can be assumed that the hypothesized aberrant homeostasis can be aggravated by new stressors in the form of infection [64], physical exertion, cognitive effort such as reading or solving mental puzzles, triggering a post-exertional malaise (PEM), comorbid conditions such as sleep disturbances [65], and other factors [26]. In people who do not develop ME/CFS or prolonged illness following an acute infection or other insult, external stressors initially cause short-term physiological changes, but the usual state of homeostatic equilibrium that operated before the insult is successfully restored.

It has been suggested that the proposed aberrant state of homeostasis could be caused by inflammation in the hypothalamus [25]. However, such hypothalamic inflammation is yet to be confirmed, although possible mechanisms have been described [66]. It should be noted that dyshomeostasis can occur through multiple disturbances in regulatory systems without any necessity for focal inflammation in the brain. Thus, hypothalamic inflammation remains one option among many for the role of inflammation in ME/CFS and PASC.

It is helpful to consider one illustrative theoretical example of how dyshomeostasis at a central systemic level could be translated into the devastating conditions of ME/CFS and PASC.

Tate et al. (2022) [5] provided a research synthesis with the following hypothesis:

*“Following activation of a systemic immune/inflammatory response to an infection or severe stress event, abnormal transport of signals or molecules into the CNS occurs through neurovascular pathways or a disrupted BBB. If the initial stressor is not resolved, this leads to fluctuating chronic neuroinflammation that sustains and controls the complex neurological symptoms of ME/CFS and long-COVID and facilitates frequent, more serious relapses in response to life stress, as evidenced from a comprehensive disruption to the cellular molecular biology and body’s physiological pathways.”*

[5] (Figure 6)

In light of the above-reviewed findings concerning the Central Homeostasis Network, widespread knock-on disruptions of functioning would be expected throughout the CNS and beyond in the pathophysiology of ME/CFS and PASC, such as those reviewed by [31].

## 8. Conclusions

(1) The pattern of symptoms for ME/CFS and PASC is highly similar. Using a psychometrically reliable and validated measure [15], the correlation in mean symptom scores across 62 symptoms is 0.902.

(2) The waxing and waning of ME/CFS and PASC symptomatology require an explanatory hypothesis that includes a mechanism that can stabilize and destabilize in unpredictable, intermittent cycles. It is suggested that ME/CFS and PASC are the consequence of central nervous system dyshomeostasis. The hypothesis receives tentative support from a variety of sources.

(3) The theory is consistent with the ‘Energy Envelope’ theory [54], research on organ network interactions [38], and hypotheses concerning molecular mechanisms of neuroinflammation in ME/CFS and PASC [5].

(4) The current theory is speculative and requires in-depth investigation before definite conclusions can be drawn. Further study of mechanisms and functional studies should improve understanding of the associations between these hypotheses and etiological factors.

## Figures and Tables

**Figure 6:**
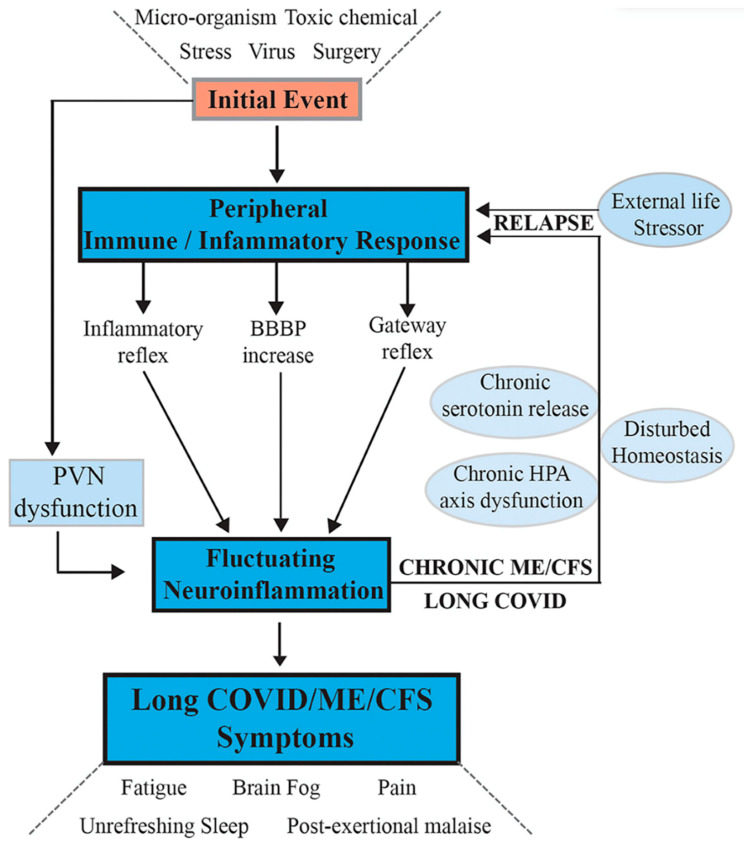
Hypothesis and model for onset of ME/CFS and its progression to a chronic sustained illness with relapse/partial recovery phases. Signaling pathways between the CNS and the periphery that maintain the illness. Following an initial external stressor event, systemic immune/inflammatory responses are activated. These are communicated to the CNS via inflammatory and gateway reflexes and possibly an increase in permeability of the BBB. Neuroinflammation is activated affecting the stress center within the PVN of the hypothalamus and leads to a wide range of neurological symptoms that feedback to the periphery via disturbance of homeostasis, and the stress-activated HPA axis that becomes dysfunctional with chronic activation. The systemic physiology and molecular homeostasis are then chronically affected through important cellular functions, such as mitochondrial energy production, metabolic activity, and a continuation of immune/inflammatory reactions. External life stressors that feed into a disturbed PVN not only maintain the ME/CFS but also act to precipitate relapses. Reproduced from [5] with permission.

**Table 1:** Proposed diagnostic criteria for ME/CFS [10].

Diagnosis requires that the patient have the following three symptoms:
A substantial reduction or impairment in the ability to engage in pre- illness levels of occupational, educational, social or personal activities, that persists for more than 6 months and is accompanied by fatigue, which is often profound, is of new or definite onset (not lifelong), is not the result of ongoing excessive exertion, and is not substantially alleviated by rest, and
2. Post-exertional malaise, * and
3. Unrefreshing sleep *
At least one of the two following manifestations is also required:
Cognitive impairment * or
2. Orthostatic intolerance

* Frequency and severity of symptoms should be assessed. The diagnosis of ME/CFS should be questioned if patients do not have these symptoms at least half of the time with moderate substantial, or severe intensity.

## Data Availability

The research for this article did not include the collection of new data.
